# Oropharyngeal and intestinal concentrations of opportunistic pathogens are independently associated with death of SARS-CoV-2 critically ill adults

**DOI:** 10.1186/s13054-022-04164-0

**Published:** 2022-10-03

**Authors:** Juliette Patrier, Khanh Villageois-Tran, Piotr Szychowiak, Stéphane Ruckly, Rémi Gschwind, Paul-Henri Wicky, Signara Gueye, Laurence Armand-Lefevre, Mehdi Marzouk, Romain Sonneville, Lila Bouadma, Marie Petitjean, Fariza Lamara, Etienne de Montmollin, Jean-Francois Timsit, Etienne Ruppé, The French COVID Cohort Study Group, Laurent Abel, Amal Abrous, Claire Andrejak, François Angoulvant, Delphine Bachelet, Marie Bartoli, Sylvie Behilill, Marine Beluze, Krishna Bhavsar, Lila Bouadma, Minerva Cervantes-Gonzalez, Anissa Chair, Charlotte Charpentier, Léo Chenard, Catherine Chirouze, Sandrine Couffin-Cadiergues, Camille Couffignal, Marie-Pierre Debray, Dominique Deplanque, Diane Descamps, Alpha Diallo, Fernanda Dias da Silva, Céline Dorival, Xavier Duval, Philippine Eloy, Vincent Enouf, Hélène Esperou, Marina Esposito-Farese, Manuel Etienne, Aline-Marie Florence, Alexandre Gaymard, Jade Ghosn, Tristan Gigante, Morgane Gilg, François Goehringer, Jérémie Guedj, Ikram Houas, Isabelle Hoffmann, Jean-Sébastien Hulot, Salma Jaafoura, Ouifiya Kafif, Antoine Khalil, Nadhem Lafhej, Cédric Laouénan, Samira Laribi, Minh Le, Quentin Le Hingrat, Soizic Le Mestre, Sophie Letrou, Yves Levy, Bruno Lina, Guillaume Lingas, Denis Malvy, France Mentré, Hugo Mouquet, Nadège Neant, Christelle Paul, Aurélie Papadopoulos, Christelle Paul, Ventzislava Petrov-Sanchez, Gilles Peytavin, Valentine Piquard, Olivier Picone, Manuel Rosa-Calatrava, Bénédicte Rossignol, Patrick Rossignol, Carine Roy, Marion Schneider, Richa Su, Coralie Tardivon, Jean-François Timsit, Sarah Tubiana, Sylvie Van Der Werf, Benoit Visseaux, Aurélie Wiedemann

**Affiliations:** 1 AP-HP, Bichat Hospital, Medical and Infectious Diseases ICU (MI2), 75018 Paris, France; 2 AP-HP, Service de Microbiologie, Hôpital Beaujon, 75018 Paris, France; 3 IAME, INSERM, Université de Paris, 75018 Paris, France; 4 AP-HP, Service de Bactériologie, Hôpital Bichat-Claude Bernard, 75018 Paris, France; 5 OUTCOME REA Research Network, IAME, INSERM, Université de Paris, 75018 Paris, France

**Keywords:** Microbiota, COVID-19, *Enterococcus*, *Candida*, *Staphylococcus aureus*, Biomarker, Resuscitation

## Abstract

**Background:**

The composition of the digestive microbiota may be associated with outcome and infections in patients admitted to the intensive care unit (ICU). The dominance by opportunistic pathogens (such *as Enterococcus*) has been associated with death. However, whether this association remains all throughout the hospitalization are lacking.

**Methods:**

We performed a single-center observational prospective cohort study in critically ill patients admitted with severe SARS-CoV-2 infection. Oropharyngeal and rectal swabs were collected at admission and then twice weekly until discharge or death. Quantitative cultures for opportunistic pathogens were performed on oropharyngeal and rectal swabs. The composition of the intestinal microbiota was assessed by 16S rDNA sequencing. Oropharyngeal and intestinal concentrations of opportunistic pathogens, intestinal richness and diversity were entered into a multivariable Cox model as time-dependent covariates. The primary outcome was death at day 90.

**Results:**

From March to September 2020, 95 patients (765 samples) were included. The Simplified Acute Physiology Score 2 *(*SAPS 2) at admission was 33 [24; 50] and a Sequential Organ Failure Assessment score (SOFA score) at 6 [4; 8]. Day 90 all-cause mortality was 44.2% (42/95). We observed that the oropharyngeal and rectal concentrations of *Enterococcus* spp., *Staphylococcus aureus* and *Candida* spp. were associated with a higher risk of death. This association remained significant after adjustment for prognostic covariates (age, chronic disease, daily antimicrobial agent use and daily SOFA score). A one-log increase in *Enterococcus* spp*.*, *S. aureus* and *Candida* spp. in oropharyngeal or rectal swabs was associated with a 17% or greater increase in the risk of death.

**Conclusion:**

We found that elevated oropharyngeal/intestinal *Enterococcus* spp. *S. aureus* and *Candida* spp. concentrations, assessed by culture, are associated with mortality, independent of age, organ failure, and antibiotic therapy, opening prospects for simple and inexpensive microbiota-based markers for the prognosis of critically ill SARS-CoV-2 patients.

**Supplementary Information:**

The online version contains supplementary material available at 10.1186/s13054-022-04164-0.

## Introduction

The human microbiota harbors a vast diversity of microorganisms (mainly bacteria), with the highest concentrations being located in the oropharyngeal and gut microbiota [1, 2]. A balanced composition of the microbiota is assumed to be associated with health, while an altered composition (referred to as dysbiosis) is associated with a broad range of intestinal and extra intestinal conditions [3]. The gut microbiota composition is dominated by strictly anaerobic bacteria, while opportunistic pathogens such as *Enterobacterales* and enterococci are subdominant [4]. Nonetheless, antibiotic exposure can alter the composition of the microbiota in promoting resistant, opportunistic pathogens which may be subsequently involved in infections [5–7]. More recently, the presence of *Enterobacterales* in rectal samples or the dominance of *Enterococcus* in patients at admission to the intensive care unit has been associated with a higher risk of death [8, 9]. Recently, the composition of the microbiota was associated with mortality at D28 in patients hospitalized in intensive care unit (ICU), with a lower diversity being associated with death [10]. Beyond colonization, we showed that the oropharyngeal and rectal concentrations of extended-spectrum beta-lactamase (ESBL)—producing *Enterobacterales*—could be a powerful tool to estimate the risk of subsequent ventilator-associated pneumonia (VAP) caused by ESBL-producing *Enterobacterales* in mechanically ventilated patients [11]. Altogether, the oropharyngeal microbiota and the gut microbiota are potential sources of knowledge from which the prognosis of critical care patients can be assessed.

More specifically, ICU patients suffering from severe presentations of COVID-19 frequently experience a high rate of nosocomial infections such as bloodstream infection (BSI) and VAP, which extend the course of ICU stay and worsen the prognosis [12, 13]. While several host risk factors for severe COVID-19 have been identified [14], factors related to the microbiota have been little studied. Nonetheless, the SARS-CoV-2 can affect the gut microbiota and induce dysbiosis. Early shotgun metagenomic sequencing analyses of fecal samples from a dozen of COVID patients showed enrichment of opportunistic pathogens and depletion of beneficial commensals [15]. Also, the composition of the intestinal microbiota has been associated with the severity of COVID [16, 17].

Our objective was to investigate the association between oropharyngeal and intestinal concentrations of opportunistic pathogens, the overall composition of the gut microbiota, and the prognosis of patients admitted to intensive care with severe forms of SARS-CoV-2 infection. Our hypothesis is that the presence of these opportunistic pathogens is a possible marker of altered gut microbiota, and that it is associated with the outcome of patients hospitalized with severe forms of COVID-19.

## Methods

### Ethics

Our ICU is part of the French COVID consortium (registered in clinicaltrials.gov NCT04262921). The French COVID scientific committee approved the NOSOCOVID ancillary study and the additional oropharyngeal and rectal samples. The study was conducted with the understanding and consent of each participant or surrogate. The French COVID received ethical clearance on February 5, 2020 by the CPP-Ile-de-France VI (ID RCB: 2020-A00256-33). The OutcomeRea™ database provided anonymous extractions of daily data collected during the ICU stays in accordance with the French law (“Commission Nationale de l’Informatique et des Libertés” #999,262).

### Population

From March to September 2020 (first wave of COVID-19), we conducted a single-center observational prospective cohort study in critically ill patients which included consecutive patients ≥ 18 years hospitalized in ICU of Bichat university hospital (Paris, France) for a proven severe SARS-CoV-2 infection. All patients admitted to the ICU with COVID-19 were included. SARS-CoV-2 infection was proven by PCR test performed in the virology laboratory of our hospital. There were no exclusion criteria, except for the patient without proven COVID-19 infection. Rectal or oropharyngeal samples were sampled twice a week during the ICU stay. Indeed, we collected oropharyngeal and rectal swabs (E-Swab, Copan, Brescia, Italy and Deltaswab, Greiner, Courtaboeuf, France) during nursing cares for each patient at admission and twice a week, on Monday and Thursday for the first month, and on Tuesday and Friday for the following months (change for logistic matters). When a patient was admitted on Monday, the next sample was on Thursday. The patient continued to be swabbed until discharge or death. The oropharyngeal swab was rubbed in the throat until it was saturated with throat secretions. As we experienced occasional shortages of E-swabs during the inclusion period, we used alternative swabs such as Regular Swab with Amies Agar Gel (Copan), Regular Swab and Liquid Amies Medium (Copan), Human DNAsa, RNAsa and DNA-free certified swabs, steriles (Deltalabs, Barcelona, Spain) in addition to brain heart infusion (BHI) broth (bioMérieux, Marcy-l’Etoile, France). The samples were then sent on a daily pace to the bacteriology laboratory.

This study is an ancillary study of the French-Cohort and is also a part of the OutcomeRea™ database allowing to extract the data anonymously. We collected general characteristics: age, sex, history, and first day of COVID symptoms. We calculated body mass index (BMI) from admission height and weight and collected the treatments (e.g., antibiotics, immunomodulatory treatments, steroids) initiated before ICU admission if the patient had been transferred from another unit. Digestive decontamination using topical antibiotics was not used. Routine biological tests at admission included complete blood count, plasma proteins (C-reactive protein, procalcitonin, interleukin-6, ferritin), d-dimers, and lactate-dehydrogenase. Regarding severity assessment, we calculated the sequential organ failure assessment (SOFA) score and the simplified acute physiology score 2 (SAPS 2) at admission, and the SOFA score on each day of hospitalization in the ICU. For death at D90, we took the last available data in our information system. Time-dependent variables were initialized to 0 and remained fixed after discharge. There were 42 (44%) deaths and 16 (17%) patients were lost to follow-up before D90.

### Culture methods

We measured the absolute concentration of pathogens in the oropharyngeal and rectal samples using the following protocol depicted in the Additional file 1: Fig. S1. For each rectal and oropharyngeal swab, 10 µL were plated onto seven agar media: (1) Columbia CNA + 5% sheep blood (bioMérieux, Marcy-l’Etoile, France) for the selection of Gram-positive bacteria, (2) Drigalski (bioMérieux) for the selection of *Enterobacterales* and non-fermenting Gram-negative bacilli, (3) Cetrimide (bioMérieux) for the selection of *Pseudomonas aeruginosa*, (4) ChromID® *S. aureus* Elite SAIDE (bioMérieux) for the selection of *Staphylococcus aureus*, (5) ChromID® ESBL (bioMérieux) for the selection of ESBL-producing Enterobacterales, (6) ChromID® CARBA (bioMérieux) for the selection of carbapenemase-producing Enterobacterales and (7) BBL™ CHROMagar™ Candida (Becton-Dicksinson, Rungis, France) for the selection of yeasts. Plates were then incubated at 37 °C for 24 to 48 h. The rest of the fluid from the rectal and throat e-swabs was stored at − 20 °C after initial viral inactivation (30 min at 37 °C) [18]. The identification of bacteria was obtained by mass spectrometry (Maldi Biotyper, Bruker Daltonics, Bremen, Germany). The quantification (expressed in colony-forming unit [CFU] per mL) was performed according to the protocol depicted in the Additional file 1: Fig. S1. As for antibiotic resistance, the following tests were performed: Alere™ PLP2A test (Abott, Rungis, France) for the detection of methicillin-resistant *S. aureus*, β LACTA™ test (Bio-Rad, Marne-La-Coquette, France) for the detection of C3G-resistant *Enterobacterales* and Xpert® CARBA (Cepheid, Sunnyvale, CA) for the detection of carbapenemase-producing Enterobacterales. A complete antibiogram (disk diffusion) was performed for *P. aeruginosa* and *Acinetobacter* sp.

### 16S rDNA sequencing

The composition of the intestinal microbiota was assessed by 16S profiling. DNA extraction of the rectal swabs was performed with the QIAamp PowerFecal Pro DNA kit (Qiagen, Courtaboeuf, France). PCR amplification of the v3-v4 segments of the 16S rDNA gene was performed using the primers proposed by Illumina (San Diego, CA). The amplicons were sequenced in paired end using a MiSeq (Illumina) device, with the target of > 10,000 read pairs per sample. The software SHAMAN was used for the analysis of the data [19]. We defined for each sample the genus richness (number of unique genera per sample) and the Shannon and the inverse Simpson indices for assessing diversity (i.e., how the different taxa are distributed). The higher the values, the more balanced the taxa are distributed.

### Statistical methods

Patients’ characteristics were expressed as number (percentage) for categorical variables and median (interquartile range [IQR]) for continuous variables. Comparisons were made using Fisher exact tests for categorical variables and Wilcoxon tests for continuous variables. All the oropharyngeal and rectal culture and microbiota variables were tested in univariate Cox models as time-dependent covariates. We also tested the impact of daily SOFA and antimicrobial use using the same method. Then, we performed a multivariable Cox model introducing the microbiota variables selected in the univariate Cox models and adjusting for prognostic covariates (age, chronic illness, daily SOFA score and daily antibiotic use). The proportionality of hazard risks for time-fixed covariates was assessed using martingale residuals. For all tests, a two-sided α of 0.05 was considered as significant. All statistical analyses were performed with SAS software, Version 9.4 (SAS Institute, Cary, NC) and R (version 3.6.3). The primary outcome was death from all causes at day 90. The secondary outcomes were death in ICU, in-hospital death, all-cause death on days 42.

## Results

### Population

Ninety-five consecutive patients were admitted in the Medical and Infectious Diseases ICU, in Bichat University hospital, (Paris, France) for a SARS-CoV-2 infection from March 19 to September 25, 2020. Patients’ characteristics are detailed in Table 1. Of note, they were mostly men (78.9%), overweight (median BMI 28.4 kg/m^2^ [24.7; 32.4]). Forty-six (48.4%) had a coexisting condition. The median time from COVID symptom onset to ICU admission was 11 days [8; 15]. During the first 48 h of ICU hospitalization, 37 patients (38.9%) received invasive ventilation, and of these, 10 (10.5%) received extracorporeal membrane oxygenation (ECMO), 15 patients (15.8%) were dialyzed, and 41 (43.2%) had vasopressors. All-cause mortality at D90 was 44.2% (42/95). The median length of stay in the ICU was 11 [6; 20] days. One third of patients (34 patients) were treated for ICU-acquired pneumonia occurring after a median of 8 days after ICU admission (IQR [6; 20]) and one quarter of patients (24 patients, 51 episodes) were treated for bloodstream infection (BSI) diagnosed 12 days in median after ICU admission (IQR [8; 15]). Last, *Enterococcus* spp., *S. aureus* and *Candida* spp. were, respectively, identified in 2, 9 and 5 episodes of BSI. Other bacteria recovered from blood cultures were (coagulase negative *Staphylococci* (n = 21), *Streptococcus* sp. (n = 5), Enterobacterales (n = 5), P *aeruginosa* (n = 4) and anaerobes (n = 2).

**Table 1:** Initial patients’ characteristics and outcomes

	N (%) [IQR]
General characteristics	
Age, y	59.7 [49.3; 66.1]
Sex, No. (%)	
Female	20 (21.1)
Body Mass Index (BMI)	28.4 [24.7; 32.4]
Medical history, No. (%)	46 (48.4)
Diabetes	25 (26.3)
Chronic heart failure	29 (30.5)
Chronic respiratory failure	14 (14.7)
Chronic renal failure	15 (15.8)
Chronic hepatic failure	2 (2.1)
Immunosuppression	23 (24.2)
Organ transplantation	11 (47.8)
Long-term steroid therapy	7 (30.4)
HIV infection	3 (13.0)
Other	13 (56.5)
Characteristics at admission	
Time from onset of the symptoms to ICU admission, days	11 [8; 15]
SAPS 2	33 [24; 50]
SOFA	6 [4; 8]
Treatment started within admission and inclusion	
Antibiotics	83 (87.4)
Cephalosporins	43 (43.9)
Macrolides	29 (29.6)
Cotrimoxazole	9 (9.2)
Ureido-penicillins	7 (7.1)
Amino-penicillins	5 (5.1)
Glycopeptides	3 (3.1)
Ciprofloxacin	3 (3.1)
Carbapenems	1 (1)
Steroids	61 (64.2)
Tocilizumab or Anakinra	28 (29.5)
Lopinavir/ritonavir	40 (42.1)
Remdesivir	15 (15.8)
Biological data at ICU admission	
Lymphocytes, /mm^3^	910 [620; 1220]
C-reactive protein, mg/L	138 [65; 218]
D-Dimer, ng/mL	1283.5 [682; 4455]
Ferritin, µg/L	1352.5 [789; 2372]
Interleukin-6, pg/mL	47.6 [20.5; 167.5]
LDH, IU/L	481 [344; 638]
Support therapy in the first 48 h	
Acute respiratory support	
Noninvasive positive pressure ventilation, high-flow nasal canula	58 (61.1)
Invasive positive pressure ventilation	27 (28.4)
Invasive positive pressure ventilation and ECMO	10 (10.5)
Renal replacement therapy	15 (15.8)
Vasopressor agent support	41 (43.2)
Medications during ICU stay	N (%)
Steroids	61 (64.2)
Aciclovir	19 (20)
Ganciclovir	7 (7.4)
Tocilizumab or Anakinra	39 (41.1)
Primary outcome	
Death at D90	42 (44.2)
Secondary outcomes	
Death in ICU	40 (42.1)
Death in hospital	42 (44.2)
Death at D42	40 (42.1)
Nosocomial infections	
Bloodstream infections	24 (25.3)
Nosocomial pneumonia*	40 (42.1)
Hospital-acquired pneumonia in non-ventilated patients	9 (9.5)
Ventilator-acquired pneumonia	34 (35.8)

SAPS 2, Simplified Acute Physiology Score; SOFA score, Sequential Organ Failure Assessment score; ECMO, Extracorporeal Membrane Oxygenation

*Nosocomial pneumonia includes hospital-acquired pneumonia in non-ventilated patients and ventilator-associated pneumonia (40 patients will develop at least one episode of nosocomial pneumonia, including 34 with at least one episode of Ventilator-acquired pneumonia and 9 with at least one episode of Hospital-acquired pneumonia in non-ventilated patients)

N = 95; no missing data

### Culture results

**Fig. 1:**
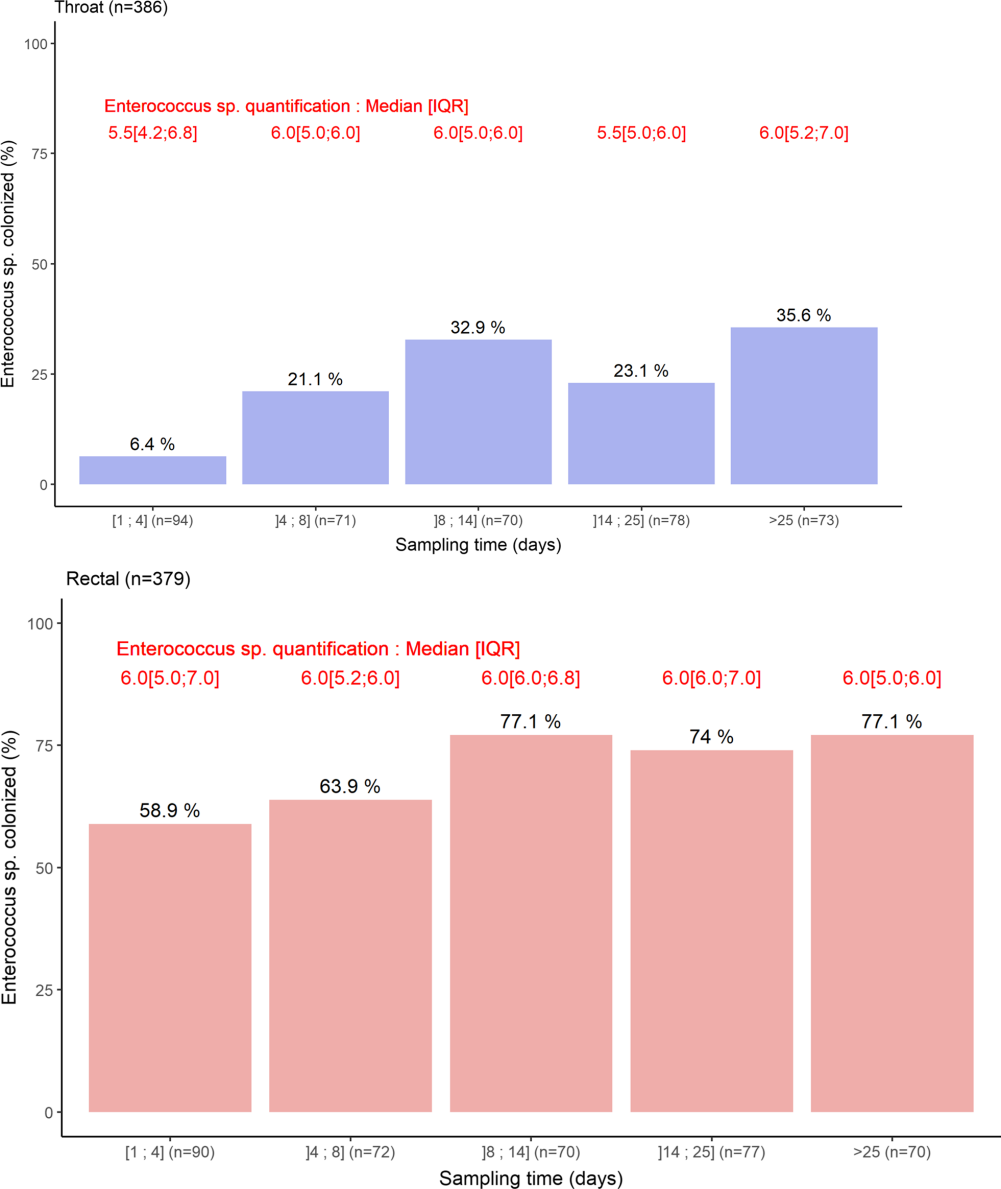
Boxplot representation of the oropharyngeal (blue) and rectal (red) concentrations of *Enterococcus* spp. according to the sampling time range. Bars indicate the percentage of positive sample and number in red indicates median (IQR) values of positive samples

We collected 765 samples (386 oropharyngeal and 379 rectal). The average time from admission to the first oropharyngeal and rectal swabs was 3 days (IQR [2; 4]). Details on per pathogen culture results are available on Additional file 1: Table S2. The crude colonization rate with *Enterococcus* spp. increased along with the days of hospitalization: 6.4% of patients were colonized by *Enterococcus* spp. in the oropharynx at admission, while 35.6% patients still hospitalized after 25 days were colonized (Fig. 1). This was less marked for the gut, with 58.9% patients colonized at admission vs. 77.1% after 25 days. In colonized patients, the median concentrations of *Enterococcus* spp. at admission were 5.5 and 6 log of colony-forming units (CFU) per mL in the throat and the gut, respectively, which remained in the same range (5–6 log CFU/mL) over time (Fig. 1**)**. Among enterococci, *E. faecalis* was the most frequent species (Additional file 1: Fig. 2).

**Fig. 2:**
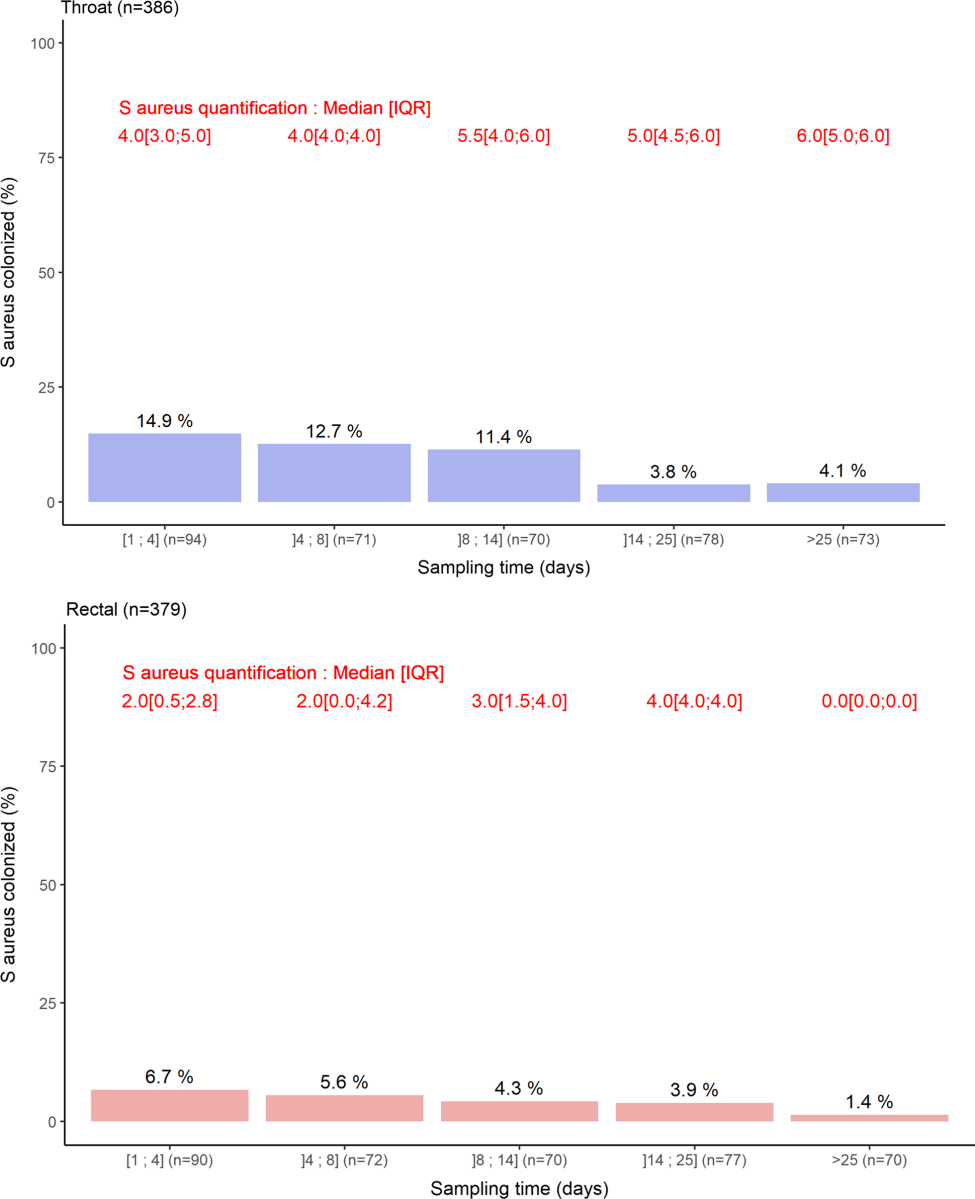
Boxplot representation of the oropharyngeal (blue) and rectal (red) concentrations of *S. aureus* according to the sampling time range. Bars indicate the percentage of positive sample and number in red indicates median (IQR) values of positive samples

The prevalences of oropharyngeal and rectal colonization by *S. aureus*, were 14.9% and 6.7%, respectively (Fig. 2). In both oropharynx and gut, the carriage prevalence dropped to 4.1% and 1.4% for patients hospitalized for 25 days or more. The median oropharyngeal and rectal concentrations at admission were both 4 log CFU per mL. Yet, for patients still colonized in the oropharynx, the concentration ranged from 5 to 6 UFC/mL, while it decreased in the gut (Fig. 2).

**Fig. 3:**
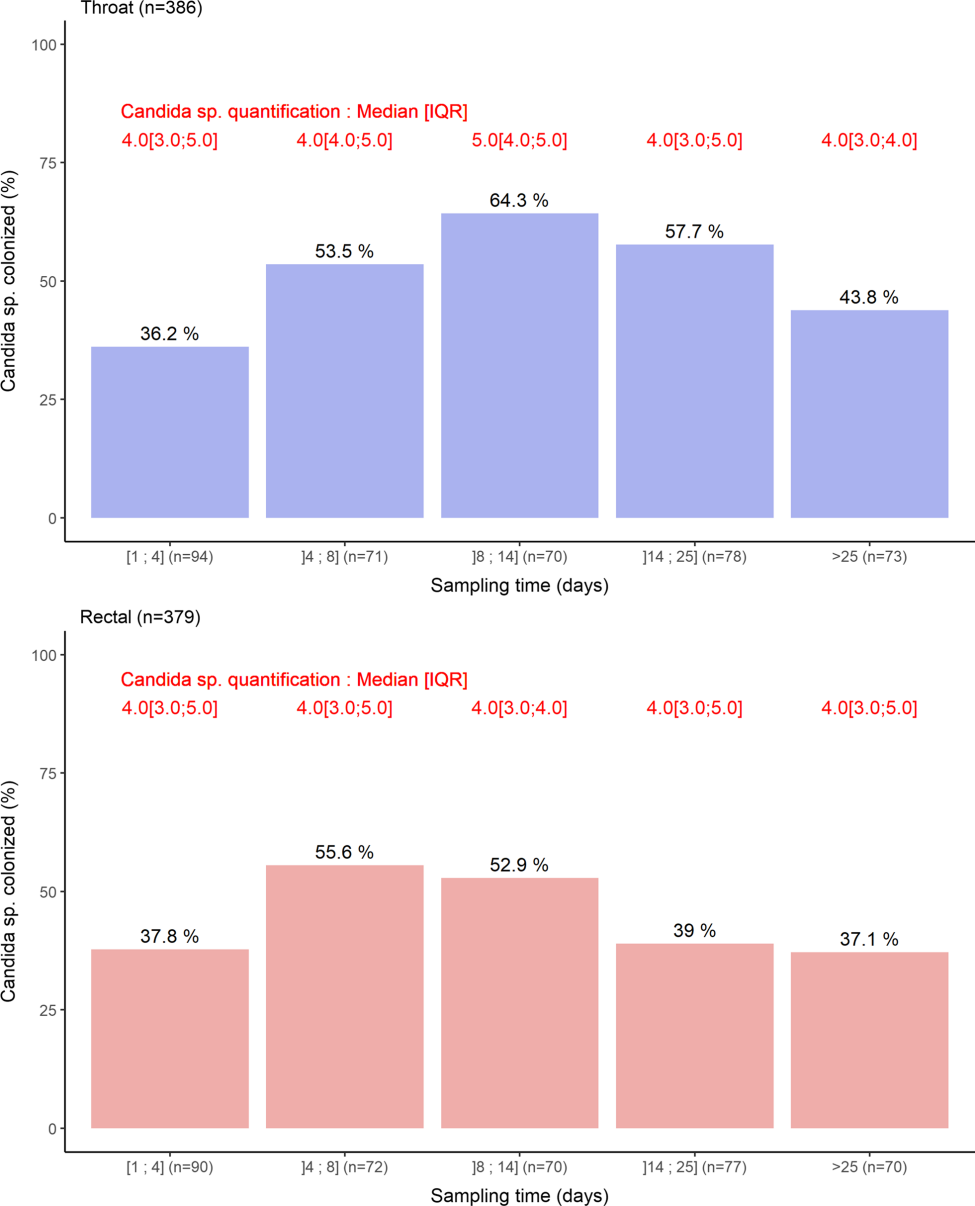
Boxplot representation of the oropharyngeal (blue) and rectal (red) concentrations of *Candida* sp. according to the sampling time range. Bars indicate the percentage of positive sample and number in red indicates median (IQR) values of positive samples

Last for *Candida* spp., the prevalences of oropharyngeal and rectal colonization were 36.2% and 37.8%, respectively (Fig. 3). The carriage prevalence tended to peak between days 8 and 14 in the oropharynx and days 4 and 14 in the gut, before returning at baseline prevalence. The median oropharyngeal and rectal concentrations at admission were both 4. *C. albicans* was the most frequent species (Additional file 1: Fig. S2).

### 16S rDNA sequencing

16S rDNA sequencing could be achieved on 339 out of 379 rectal swabs: Two were missing and 38 were excluded due to low quality (less than 10.000 reads obtained). At admission, average richness (genus level), Shannon and inverse Simpson indices were 30, 2.5 and 8.0, respectively (Additional file 1: Table S1). In patients colonized with *Enterococcus* spp. at admission, we found an association between richness and diversity (inverse Simpson indices) and the gut concentrations of *Enterococcus* spp. (Additional file 1: Fig. S3). However, in patients colonized with *Candida* spp., such association was not found (Additional file 1: Fig. S3).

### Association with mortality

In the univariate analysis (Table 2), the serial absolute abundance of *Enterococcus* spp., *S aureus*, and *Candida* spp., either in the oropharynx or in the rectum, was strongly associated with mortality at D90. Of note, such association was not found with other opportunistic pathogens (Enterobacterales, *P. aeruginosa*, ESBL-producing Enterobacterales). In multivariable analysis adjusted for age, chronic diseases, daily SOFA score, and daily use of anti-infective agents, a high oropharyngeal or rectal colonization with *Enterococcus* spp.*, S. aureus* and *Candida* spp. was again found to be independently associated with mortality at D90. Of note, neither intestinal richness nor diversity as determined by 16S rDNA sequencing was associated with mortality (Table 2).

**Table 2:** Univariate analysis of day 90 mortality*

	HR	95% CI		*p*
Oropharynx (time-dependent variables)				
* Enterococcus* spp*.*	1.268	1.172	1.371	< 0.0001
Enterobacterales	0.964	0.862	1.078	0.5231
* Pseudomonas aeruginosa*	0.950	0.774	1.165	0.6202
ESBL-producing Enterobacterales	0.992	0.790	1.246	0.9439
* Candida* spp*.*	1.170	1.065	1.285	0.0010
* S. aureus*	1.211	1.081	1.358	0.0010
Rectal (time-dependent variables)				
* Enterococcus* spp*.*	1.198	1.095	1.310	< 0.0001
Enterobacterales	0.942	0.870	1.020	0.1416
* Pseudomonas aeruginosa*	0.980	0.867	1.108	0.7448
ESBL-producing Enterobacterales	0.914	0.791	1.057	0.2263
* Candida* spp*.*	1.198	1.077	1.332	0.0009
* S. aureus*	1.309	1.088	1.573	0.0042
16S/rectal (time-dependent variables)				
Richness	1.000	1.000	1.000	0.1407
Shannon index	1.006	0.733	1.380	0.9696
Bacteroides***	2.501	0.530	11.801	0.2467
Enterococcus***	5.300	2.034	13.814	0.0006
Finegoldia***	0.013	0.001	0.286	0.0058
Enterobacterales***	1.224	0.145	10.333	0.8524
Firmicutes/Bacteroidetes ratio	0.998	0.994	1.001	0.1835
Antimicrobial therapy (time-dependent variables)				
Antifungal treatment active against Candida#	4.298	2.662	6.940	< 0.0001
Antibiotic treatment active against Enterococcus spp.#	5.998	3.742	9.614	< 0.0001
Fluoroquinolones	0.947	0.339	2.645	0.9168
Daptomycin/Glycopeptides/Linezolid	7.300	4.564	11.677	< 0.0001
Antibiotic treatment active against anaerobic bacteria#	5.895	3.811	9.118	< 0.0001
General characteristics and treatment during the 1st 48 h of ICU admission				
Age	1.033	1.006	1.061	0.0172
Female	1.184	0.566	2.477	0.6529
Obesity	0.658	0.345	1.255	0.2040
Diabetes	1.666	0.876	3.169	0.1199
Chronic diseases	1.776	0.958	3.291	0.0681
SAPS 2*	1.012	1.000	1.025	0.0476
SOFA score*	1.192	1.087	1.306	0.0002
C-reactive protein*	1.002	1.000	1.005	0.0884
Steroids*	1.387	0.709	2.712	0.3389
Renal replacement therapy**	1.674	0.800	3.502	0.1710
Vasopressors**	1.591	0.868	2.917	0.1328
Ventilation status**				
Mechanical ventilation with PEEP > 10 cmH2O	1.992	1.035	3.834	0.0391
ECMO	1.897	0.757	4.755	0.1719
Antibiotics**	2.225	0.687	7.207	0.1821

SAPS 2, Simplified Acute Physiology Score; SOFA score, Sequential Organ Failure Assessment score; OUT, Operational Taxonomic Unit; B/F ratio, Bacteroidetes/Firmicutes ratio; ECMO, Extracorporeal Membrane Oxygenation; HR, Hazard ratio on the final model; 95% CI, 95% confidence interval of the adjusted HR

*At admission; **During the first 48 h in ICU; ***Abundance

^#^Antifungal treatment active against Candida includes intravenous (IV) polyenes, azoles and IV candins. Antibiotic treatment active against Enterococcus/MRSA includes vancomycin, daptomycin and oxazolidinones. Antibiotic treatment active against anaerobic bacteria includes nitro-imidazoles, imipenem, meropenem, clindamycin, piperacillin/tazobactam and amoxicillin/clavulanate

NB: only one patient was colonized with carbapenemase-producing Enterobacterales. The variable was not tested statistically

We found that certain anti-infective agents were also strongly related to the primary outcome. Indeed, in univariate analysis and confirmed in the multivariable model, receiving antifungal treatment active against *Candida*, or antibiotic treatment active against anaerobic bacteria, *Enterococcus* spp. and methicillin-resistant *S. aureus* (MRSA) was strongly associated with mortality (Tables 2 and 3, and Additional file 1: Tables S3, S4).

**Table 3:** Adjusted impact of *Enterococcus* spp., *S. aureus* and *Candida* spp. oropharyngeal and rectal abundances on day 90 mortality (combined analyses)

	HR	95% CI		*p*
**Oropharynx**				
* Enterococcus* spp*.* Quantitative (log)	1.100	1.010	1.197	0.0281
Antibiotic treatment active against *Enterococcus*/MRSA#	2.872	1.704	4.841	< 0.0001
* Candida* spp*.* Quantitative (log)	1.183	1.065	1.313	0.0017
Antifungal treatment active against *Candida* spp.#	1.499	0.846	2.655	0.1650
* S. aureus* quantitative (log)	1.265	1.112	1.440	0.0004
Antibiotic treatment active against anaerobic bacteria#	2.240	1.343	3.737	0.0020
Age*	1.026	1.004	1.049	0.0230
Chronic diseases**	2.047	1.273	3.291	0.0031
Daily SOFA score*	1.172	1.105	1.243	< 0.0001
**Rectal**				
* Enterococcus* spp*.* quantitative (log)	1.156	1.052	1.270	0.0026
Antibiotic treatment active against *Enterococcus*/MRSA#	2.253	1.296	3.915	0.0040
* Candida* spp*.* quantitative (log)	1.182	1.059	1.320	0.0029
Antifungal treatment active against *Candida* spp.#	0.991	0.558	1.759	0.9740
Antibiotic treatment active against anaerobic bacteria#	2.842	1.706	4.733	< 0.0001
* S. aureus* quantitative (log)	1.470	1.207	1.789	0.0001
Age*	1.030	1.008	1.053	0.0078
Chronic diseases**	2.071	1.267	3.386	0.0037
Daily SOFA score*	1.226	1.157	1.299	< 0.0001

SOFA score, Sequential Organ Failure Assessment score, assesses daily

*HR per one point increase in variables

**Using Knaus definitions [30]; HR, Hazard ratio on the final model; 95% CI, 95% confidence interval of the adjusted HR

#Antifungal treatment active against Candida includes: IV polyenes, azoles and IV candins. Antibiotic treatment active against Enterococcus/MRSA includes: vancomycin, daptomycin and oxazolidinones. Antibiotic treatment active against anaerobic bacteria includes nitro-imidazoles, imipenem, meropenem, clindamycin, piperacillin/tazobactam and amoxicillin/clavulanate

## Discussion

We showed a direct relationship between the serial concentrations of *Enterococcus* spp*., S. aureus* and *Candida* spp. in the gut and oropharynx of severe COVID-19 patients and mortality at D90. This relationship persisted after adjustment for mortality risk factors, daily severity of organ failure, and daily use of anti-infective therapy during the ICU stay. Considering the daily use of antibacterial agents or the richness and diversity of the gut microbiota did not add significant prognostic information.

In a previous study, Freedberg et al. showed that a dominance of *Enterococcus* spp. at ICU admission was associated with mortality [9]. Another study from Agudelo-Ochoa et al. similarly reported that in ICU patients, the abundance of intestinal *Enterococcus* spp. was higher in sepsis patients who died compared to sepsis patients who survived [20]. In COVID-19 patients, high intestinal concentrations of *Enterococcus* spp. have been associated with severe presentations [16, 17]. Recently, our group observed a strong association between the diversity of the intestinal microbiota of ICU patients and the relative abundance of *Enterococcus* spp., in that a low diversity was associated with high relative abundances of *Enterococcus* spp. [21]. Altogether, these observations support that the quantification of *Enterococcus* spp. could be a potential biomarker reflecting dysbiosis. Furthermore, in patients who received allogeneic hematopoietic cell transplantation, Stein-Thoeringer et al. showed that the type of diet (especially lactose intake) could influence the abundance of *Enterococcus* spp., which increase was associated with a higher risk of graft-versus-host disease (GvHD) [22]. In all, data supporting that *Enterococcus* spp. could be a biomarker of interest for ICU patients is accumulating, and simple measurement methods are expected. In this perspective, we used a simple, cheap culture method to measure the oropharyngeal and intestinal concentrations of *Enterococus* spp.. Like *Enterococcus* spp., *Candida* spp. has also been observed to be prevalent in ICU patients [23], but alongside with *S. aureus*, had not been associated with a poor outcome.

Our analysis also revealed that the association between the concentrations of *Enterococcus* spp., *S. aureus* and *Candida* spp. and D90 mortality persisted even after considering the exposure to antifungal and antibacterial agents active against these species. One possible hypothesis is that when the gut microbiota is altered, *S. aureus*, *Enterococcus* spp. and *Candida* spp. may not be involved in infectious processes per se which could respond to antibiotics, but rather promote inflammatory reactions and multi-organ failure. Indeed, in mouse models, *Candida* infection acts synergistically with *S. aureus* to promote the pro-inflammatory response [24, 25]. Using a mouse model of properly resuscitated peritonitis, Panpetch et al. found that *Candida* ingestion decreased animal survival and increased pro-inflammatory cytokine production [26, 27]. We also found that the administration of treatment active against anaerobic bacteria, *Enterococcus*/MRSA and antifungal drugs active against *Candida* spp. was linked to a poor prognosis. One hypothesis is that such administration fuels dysbiosis and thereby promotes the emergence of opportunistic pathogens. Another hypothesis is that the use of such drugs may reflect the suspicion or occurrence of infections themselves associated with a poor prognosis.

Our study has limitations. First, it is a single-center study and our results may not apply in other settings. Also, our population was homogeneous in that we only included severe COVID-19 patients. Our results should be replicated in other ICU populations and in non-COVD-19 severe patients. While stool remains the reference for the analysis of the gut microbiota, several studies have shown that 16S rDNA sequencing from rectal swabs is a suitable alternative. Swabs reliably reproduce the bacterial composition of the stool microbiota at alpha and beta diversity levels [28]. Obtaining stool in intensive care units is challenging, and in routine practice, rectal swabs are commonly used. In addition, despite the fact that we used several different types of swabs during the study, we believe that the impact on the culture or 16S rDNA sequencing results should be minimal as storage conditions were optimal, kept at room temperature for less than 24 h then stored at − 20 °C [29]. The samples were taken twice a week for practical reasons. Though, time from admission to sample was not perfectly identical between patients and may have introduced at random inter-individual variability. Last, we found that the oropharyngeal and gut concentrations of *Enterococcus* spp., *S. aureus* and *Candida* spp. were significantly associated with to the risk of death, the underlying mechanisms remain hypothetical.

In conclusion, we observed that in severe critically ill SARS-CoV2 patients, the oropharyngeal and intestinal concentrations of *Enterococcus* spp*.*, *S. aureus* and *Candida* spp*.* as assessed by simple quantitative cultures were associated with the mortality, even when taking into account risk factors of mortality and evolution of the main organ failures. Our results open perspectives for simple, cheap microbiota-based markers for the prognosis of critically ill patients.

## Supplementary Information

**Additional file 1: Table S1**. Result of the initial 16S rDNA sequencing in rectal samples. **Table S2**. Per pathogen rectal and oropharyngeal culture positivity, time to first positivity and abundance at first positivity. **Table S3**. Adjusted impact of abundance of *Enterococcus* spp., *S. aureus* and *Candida* spp. in oropharynx and rectum on day 90 mortality. (one adjusted model for each). **Table S4**. Univariate analysis of day 90 mortality* comparing survivors versus descedents. **Fig S1**. Quantitative culturing on agar plates. CFU: colony-forming unit. **Fig S2**. Pie chart of the distribution of *Enterococcus* spp. and *Candida* spp. species obtained by culture of all the rectal swabs (A) and oropharyngeal (B) swabs. **Fig S3**. Dot plots of the intestinal *Enterococcus* spp. and *Candida* spp. with regards to intestinal richness (genus level), Shannon and inverse Simpson indices. The Pearson correlation test was used (with the intestinal concentrations being considered as continuous variables).

## Data Availability

The datasets used and/or analyzed during the current study are available from the corresponding author on reasonable request. The reads have been deposited at the NCBI SRA (bioproject number PRJNA860047).
