# Supplementary material for: Oropharyngeal and intestinal concentrations of opportunistic pathogens are independently associated with death of SARS-CoV-2 critically ill adults
Source: Crit Care. 2022 Oct 3;26:300. doi: 10.1186/s13054-022-04164-0 (PMC9527714; doi:10.1186/s13054-022-04164-0)
Supplement: Supplementary file 1 — Additional file 1: Table S1. Result of the initial 16S rDNA sequencing in rectal samples. Table S2. Per pathogen rectal and oropharyngeal culture positivity, time to first positivity and abundance at first positivity. Table S3. Adjusted impact of abundance of Enterococcus spp., S. aureus and Candida spp. in oropharynx and rectum on day 90 mortality. (one adjusted model for each). Table S4. Univariate analysis of day 90 mortality* comparing survivors versus descedents. Fig S1. Quantitative culturing on agar plates. CFU: colony-forming unit. Fig S2. Pie chart of the distribution of Enterococcus spp. and Candida spp. species obtained by culture of all the rectal swabs (A) and oropharyngeal (B) swabs. Fig S3. Dot plots of the intestinal Enterococcus spp. and Candida spp. with regards to intestinal richness (genus level), Shannon and inverse Simpson indices. The Pearson correlation test was used (with the intestinal concentrations being considered as continuous variables). [file 13054_2022_4164_MOESM1_ESM.docx]

**Oropharyngeal and intestinal concentrations of opportunistic pathogens are independently associated with death of SARS-CoV-2 critically ill adults**

Juliette Patrier^1^, Khanh Villageois-Tran^2,3^, Piotr Szychowiak^1,3^, Stéphane Ruckly^3^, Rémi Gschwind^3^, Paul-Henri Wicky^1^, Signara Gueye^4^, Laurence Armand-Lefevre^3,4^, Mehdi Marzouk^1^, Romain Sonneville^1,3^, Lila Bouadma^1,3^, Fariza Lamara^1^, Etienne De Montmollin^1,3^, Jean-Francois Timsit^1,3^, Etienne Ruppé^3,4^ , and the *French COVID cohort study group**.

1 AP-HP, Service de Réanimation Médicale et Infectieuse, Hôpital Bichat-Claude Bernard, F-75018 Paris, France

2 AP-HP, Service de Microbiologie, Hôpital Beaujon, F-75018 Paris, France

3 INSERM, Université de Paris, IAME, F-75018 Paris, France.

4 AP-HP, Service de Bactériologie, Hôpital Bichat-Claude Bernard, F-75018 Paris, France

* Laurent ABEL, Amal ABROUS, Claire ANDREJAK, François ANGOULVANT, Delphine BACHELET, Marie BARTOLI, Sylvie BEHILILL, Marine BELUZE, Krishna BHAVSAR, Lila BOUADMA, Minerva CERVANTES-GONZALEZ, Anissa CHAIR, Charlotte CHARPENTIER, Léo CHENARD, Catherine CHIROUZE, Sandrine COUFFIN-CADIERGUES, Camille COUFFIGNAL, Marie-Pierre DEBRAY, Dominique DEPLANQUE, Diane DESCAMPS, Alpha DIALLO, Fernanda DIAS DA SILVA, Céline DORIVAL, Xavier DUVAL, Philippine ELOY, Vincent ENOUF, Hélène ESPEROU, Marina ESPOSITO-FARESE, Manuel ETIENNE, Aline-Marie FLORENCE, Alexandre GAYMARD, Jade GHOSN, Tristan GIGANTE, Morgane GILG, François GOEHRINGER, Jérémie GUEDJ, Ikram HOUAS, Isabelle HOFFMANN, Jean-Sébastien HULOT, Salma JAAFOURA, Ouifiya KAFIF, Antoine KHALIL, Nadhem LAFHEJ, Cédric LAOUÉNAN, Samira LARIBI, Minh LE, Quentin LE HINGRAT, Soizic LE MESTRE, Sophie LETROU, Yves LEVY, Bruno LINA, Guillaume LINGAS, Denis MALVY, France MENTRÉ, Hugo MOUQUET, Nadège NEANT, Christelle PAUL, Aurélie PAPADOPOULOS, Christelle PAUL, Ventzislava PETROV-SANCHEZ, Gilles PEYTAVIN, Valentine PIQUARD, Olivier PICONE, Manuel ROSA-CALATRAVA, Bénédicte ROSSIGNOL, Patrick ROSSIGNOL, Carine ROY, Marion SCHNEIDER, Richa SU, Coralie TARDIVON, Jean-François TIMSIT, Sarah TUBIANA, Sylvie VAN DER WERF, Benoit VISSEAUX, Aurélie WIEDEMANN

**Additional file 1: Table S1**. Result of the initial 16S rDNA sequencing in rectal samples.

| ***16S / rectal*** | N=95 |
| --- | --- |
| Delay between 1st sample and ICU admission (days) | **2[1;3]** |
| Shannon index | 2.5 [2.1 ; 2.9] |
| Richness | 30 [22 ; 43] |
| Inv Simpson | 8 [5.1 ; 11.1] |
| Specific bacterial relative abundances |  |
| *Faecalibacterium* | 0.0022 [0.0001 ; 0.0187] |
| *Bacteroides* | 0.0737 [0.0191 ; 0.152] |
| *Bifidobacterium* | 0.0002 [0 ; 0.0046] |
| *Corynebacteriun* | 0.0128 [0.0021 ; 0.0569] |
| *Staphylococcus* | 0.0017 [0.0003 ; 0.0147] |
| *Enterococcus* | 0.0036 [0.0001 ; 0.0898] |
| *Finegoldia* | 0.0629 [0.0217 ; 0.1478] |
| Enterobacterales | 0.0011 [0.0001 ; 0.0143] |
| Firmicutes/Bacteroidetes ratio | 2.7 [1.6 ; 5.4] |

**Additional file 1: Table S2**. Per pathogen rectal and oropharyngeal culture positivity, time to first positivity and abundance at first positivity.

| **Oropharynx** |  |  |
| --- | --- | --- |
| *Enterococcus* spp. (n,%) | n (%) | 39 (41.1) |
| Time to positivity (days) | Median [IQR] | 9 [6 ; 15] |
| First semi-quantitative count (log10) | Median [IQR] | 6 [4 ; 6] |
| Enterobacterales | n (%) | 38 (40) |
| Time to positivity (days) | Median [IQR] | 5 [4 ; 9] |
| First semi-quantitative count (log10) | Median [IQR] | 4.5 [3 ; 6] |
| *Pseudomonas* spp. | n (%) | 14 (14.7) |
| Time to positivity (days) | Median [IQR] | 8.5 [4 ; 17] |
| First semi-quantitative count (log10) | Median [IQR] | 3.5 [3 ; 5] |
| *A. baumannii* | n (%) | 3 (3.2) |
| Time to positivity (days) | Median [IQR] | 11 [2 ; 23] |
| First semi-quantitative count (log10) | Median [IQR] | 4 [2 ; 7] |
| ESBL-producing Enterobacterales | n (%) | 8 (8.4) |
| Time to positivity (days) | Median [IQR] | 5.5 [4 ; 12] |
| First semi-quantitative count (log10) | Median [IQR] | 5.5 [4 ; 6] |
| Carbapenemase-producing Enterobacterales | n (%) | 1 (1.1) |
| Time to positivity (days) | Median [IQR] | 5 [5 ; 5] |
| First semi-quantitative count (log10) | Median [IQR] | 6 [6 ; 6] |
| *Candida* spp. | n (%) | 58 (61.1) |
| Time to positivity (days) | Median [IQR] | 4 [2 ; 6] |
| First semi-quantitative count (log10) | Median [IQR] | 4 [3 ; 5] |
| *S. aureus* | n (%) | 21 (22.1) |
| Time to positivity (days) | Median [IQR] | 4 [2 ; 5] |
| First semi-quantitative count (log10) | Median [IQR] | 4 [3 ; 5] |
| **Rectal sample** |  |  |
| *Enterococcus* spp. (n,%) | n (%) | 74 (77.9) |
| Time to positivity (days) | Median [IQR] | 4 [2 ; 5] |
| First semi-quantitative count (log10) | Median [IQR] | 6 [5 ; 7] |
| Enterobacterales | n (%) | 75 (78.9) |
| Time to positivity (days) | Median [IQR] | 3 [2 ; 5] |
| First semi-quantitative count (log10) | Median [IQR] | 5 [4 ; 6] |
| *Pseudomonas* spp. | n (%) | 24 (25.3) |
| Time to positivity (days) | Median [IQR] | 4.5 [3 ; 8] |
| First semi-quantitative count (log10) | Median [IQR] | 4.5 [3 ; 6] |
| *A. baumannii* | n (%) | 6 (6.3) |
| Time to positivity (days) | Median [IQR] | 17 [2 ; 27] |
| First semi-quantitative count (log10) |  | 3.5 [2 ; 4] |
| ESBL-producing Enterobacterales | n (%) | 20 (21.1) |
| Time to positivity (days) | Median [IQR] | 4 [2 ; 5.5] |
| First semi-quantitative count (log10) | Median [IQR] | 5 [4 ; 6] |
| Carbapenemase-producing Enterobacterales | n (%) | 1 (1.1) |
| Time to positivity (days) | Median [IQR] | 5 [5 ; 5] |
| First semi-quantitative count (log10) | Median [IQR] | 4 [4 ; 4] |
| *Candida* spp. | n (%) | 59 (62.1) |
| Time to positivity (days) | Median [IQR] | 5 [2 ; 6] |
| First semi-quantitative count (log10) | Median [IQR] | 4 [3 ; 5] |
| *S. aureus* | n (%) | 14 (14.7) |
| Time to positivity (days) | Median [IQR] | 5.5 [2 ; 11] |
| First semi-quantitative count (log10) | Median [IQR] | 4 [3 ; 5] |

**Additional file 1: Table S3**. Adjusted impact of abundance of *Enterococcus* spp., *S. aureus* and *Candida* spp. in oropharynx and rectum on day 90 mortality. (one adjusted model for each)

|  | **HR** | **95% CI** | | ***p value*** |
| --- | --- | --- | --- | --- |
| **OROPHARYNX** | | | | |
| ***Enterococcus* spp. model** |  |  |  |  |
| Age* | **1.031** | 1.009 | 1.055 | 0.0068 |
| Chronic disease** | **1.506** | 0.958 | 2.367 | 0.0760 |
| Daily SOFA score* | **1.210** | 1.152 | 1.272 | <.0001 |
| *Enterococcus* spp*.* quantitative (log10) | **1.146** | 1.056 | 1.244 | 0.0011 |
| Antibiotic treatment active against *Enterococcus*/MRSA# spp. # | **3.376** | 2.071 | 5.504 | <.0001 |
| ***Candida* model** |  |  |  |  |
| Age* | **1.031** | 1.009 | 1.052 | 0.0047 |
| Chronic disease** | **1.628** | 1.036 | 2.557 | 0.0344 |
| Daily SOFA score* | **1.233** | 1.171 | 1.299 | <.0001 |
| *Candida* spp*.* quantitative (log10) | **1.184** | 1.074 | 1.306 | 0.0007 |
| Antifungal treatment active against *Candida* spp. # | **2.284** | 1.344 | 3.880 | 0.0023 |
| **S. aureus model** |  |  |  |  |
| Age* | **1.028** | 1.006 | 1.051 | 0.0141 |
| Chronic disease** | **1.831** | 1.138 | 2.945 | 0.0127 |
| Daily SOFA score* | **1.214** | 1.156 | 1.274 | <.0001 |
| *S. aureus* quantitative (log10) | **1.256** | 1.106 | 1.425 | 0.0004 |
| Antibiotic treatment active against *Enterococcus*/MRSA# | **4.282** | 2.613 | 7.018 | <.0001 |
| **RECTAL** |  |  |  |  |
| ***Enterococcus* spp. model** |  |  |  |  |
| Age* | **1.036** | 1.013 | 1.060 | 0.0020 |
| Chronic disease** | **1.492** | 0.938 | 2.373 | 0.0909 |
| Daily SOFA score* | **1.242** | 1.182 | 1.304 | <.0001 |
| *Enterococcus* spp*.* quantitative (log10) | **1.161** | 1.058 | 1.274 | 0.0017 |
| Antibiotic treatment active against *Enterococcus*/MRSA# | **2.575** | 1.535 | 4.318 | 0.0003 |
| ***Candida* model** |  |  |  |  |
| Age* | **1.032** | 1.010 | 1.054 | 0.0049 |
| Chronic disease** | **1.648** | 1.030 | 2.638 | 0.0373 |
| Daily SOFA score* | **1.249** | 1.186 | 1.315 | <.0001 |
| *Candida* spp*.* quantitative (log) | **1.180** | 1.060 | 1.314 | 0.0026 |
| Antifungal treatment active against *Candida* spp.# | **1.456** | 0.843 | 2.515 | 0.1784 |
| **S. aureus model** |  |  |  |  |
| Age* | **1.036** | 1.013 | 1.059 | 0.0019 |
| Chronic disease** | **1.441** | 0.906 | 2.291 | 0.1224 |
| Daily SOFA score* | **1.254** | 1.192 | 1.320 | <.0001 |
| *S. aureus* quantitative (log10) | **1.505** | 1.241 | 1.824 | <.0001 |
| Antibiotic treatment active against *Enterococcus*/MRSA # | **3.612** | 2.141 | 6.094 | <.0001 |

*Legends: SOFA score: Sequential Organ Failure Assessment score assesses daily. (*) HR per one point increase of variables. (**) using Knaus definitions; HR: Hazard ratio on the final model; 95% CI: 95% confidence interval of the adjusted HR.*

*# Antifungal treatment active against Candida spp. includes: IV polyenes, azoles and IV candins*

*#Antibiotic treatment includes: glycopeptides, daptomycin, oxazolidinones*

**Additional file 1: Table S4**. Univariate analysis of day 90 mortality* comparing survivors versus descedents

|  | **D90 survivors (n=53)** | **D90 decedents (n=42)** | **HR** | **95% CI** | | ***p*** |
| --- | --- | --- | --- | --- | --- | --- |
| ***Oropharynx (time-dependent variables)*** | | | | | | |
| Enterococcus spp. Colonisation 48h, n (%) | 0 | 4 (9.5) |  |  |  |  |
| Quantification, median [IQR] |  | 5.5 [4.5 ; 6.5] | **1.268** | 1.172 | 1.371 | <.0001 |
| Enterobacterales Colonisation 48h, n (%) | 1 (1.9) | 2 (4.8) | **0.964** | 0.862 | 1.078 | 0.5231 |
| Quantification, median [IQR] | 5 [5 ; 5] | 6 [4 ; 8] |  |  |  |  |
| Pseudomonas aeruginosa Colonisation 48h, n (%) | 0 | 1 (2.4) | **0.950** | 0.774 | 1.165 | 0.6202 |
| Quantification, median [IQR] |  | 7 [7 ; 7] |  |  |  |  |
| ESBL-producing Enterobacterales Colonisation 48h, n (%) | 1 (1.9) | 0 | **0.992** | 0.790 | 1.246 | 0.9439 |
| Quantification, median [IQR] | 5 [5 ; 5] |  |  |  |  |  |
| Candida spp. Colonisation 48h, n (%) | 6 (11.3) | 11 (26.2) | **1.170** | 1.065 | 1.285 | 0.0010 |
| Quantification, median [IQR] | 4 [3 ; 5] | 4 [3 ; 5] |  |  |  |  |
| S. aureus Colonisation 48h, n (%) | 4 (7.5) | 5 (11.9) | **1.211** | 1.081 | 1.358 | 0.0010 |
| Quantification, median [IQR] | 3.5 [2.5 ; 5.5] | 4 [4 ; 5] |  |  |  |  |
| ***Rectal (time-dependent variables)*** | | | | | | |
| Enterococcus spp. Colonisation 48h, n (%) | 9 (17) | 15 (35.7) | **1.198** | 1.095 | 1.310 | <.0001 |
| Quantification, median [IQR] | 6 [5 ; 7] | 6 [6 ; 7] |  |  |  |  |
| Enterobacterales Colonisation 48h, n (%) | 13 (24.5) | 14 (33.3) | **0.942** | 0.870 | 1.020 | 0.1416 |
| Quantification, median [IQR] | 5 [4 ; 6] | 6 [4 ; 7] |  |  |  |  |
| Pseudomonas aeruginosa Colonisation 48h, n (%) | 1 (1.9) | 3 (7.1) | **0.980** | 0.867 | 1.108 | 0.7448 |
| Quantification, median [IQR] | 6 [6 ; 6] | 5 [3 ; 6] |  |  |  |  |
| ESBL-producing Enterobacterales Colonisation 48h, n (%) | 3 (5.7) | 4 (9.5) | **0.914** | 0.791 | 1.057 | 0.2263 |
| Quantification, median [IQR] | 6 [2 ; 6] | 6 [5 ; 6.5] |  |  |  |  |
| Candida spp. Colonisation 48h, n (%) | 9 (17) | 8 (19) | **1.198** | 1.077 | 1.332 | 0.0009 |
| Quantification, median [IQR] | 4 [3 ; 4] | 3.5 [3 ; 5.5] |  |  |  |  |
| S. aureus Colonisation 48h, n (%) | 2 (3.8) | 3 (7.1) | **1.309** | 1.088 | 1.573 | 0.0042 |
| Quantification, median [IQR] | 4 [4 ; 4] | 4 [3 ; 5] |  |  |  |  |
| ***16S / rectal (time-dependent variables)*** | | | | | | |
| Richness 48h (n=36), n (%) | 9436 [9169 ; 10500] | 9651 [9183 ; 10511] | **1.000** | 1.000 | 1.000 | 0.1407 |
| Shannon index 48h (n=36), n (%) | 2.4 [1.9 ; 2.8] | 2.3 [2 ; 2.9] | **1.006** | 0.733 | 1.380 | 0.9696 |
| Bacteroides*** 48h (n=36), n (%) | 0.05 [0.01 ; 0.09] | 0.04 [0.02 ; 0.15] | **2.501** | 0.530 | 11.801 | 0.2467 |
| Enterococcus*** 48h (n=36), n (%) | 0 [0 ; 0.09] | 0.01 [0 ; 0.1] | **5.300** | 2.034 | 13.814 | 0.0006 |
| Finegoldia*** 48h (n=36), n (%) | 0.06 [0.01 ; 0.21] | 0.08 [0.01 ; 0.15] | **0.013** | 0.001 | 0.286 | 0.0058 |
| Enterobacterales*** 48h (n=36), n (%) | 0.001 [0 ; 0.006] | 0 [0 ; 0.004] | **1.224** | 0.145 | 10.333 | 0.8524 |
| Firmicutes/Bacteroicidetes ratio 48h (n=36), n (%) | 3.7 [2 ; 7.7] | 2.4 [1.8 ; 3.9] | **0.998** | 0.994 | 1.001 | 0.1835 |
| ***Antimicrobial therapy (time-dependent variables)*** | | | | | | |
| Antifungal treatment active against Candida # 48h, n(%) | 1 (1.9) | 1 (2.4) | 4.298 | 2.662 | 6.940 | *<.0001* |
| Antibiotic treatment active against Enterococcus spp.# 48h, n(%) | 0 | 1 (2.4) | 5.998 | 3.742 | 9.614 | *<.0001* |
| Fluoroquinolones #48h, n(%) | 1 (1.9) | 0 | 0.947 | 0.339 | 2.645 | 0.9168 |
| Daptomycin/Glycopeptide/Linezolid 48h#, n(%) | 0 | 1 (2.4) | 7.300 | 4.564 | 11.677 | *<.0001* |
| Antibiotic treatment active against anaerobic bacteria # 48h, n(%) | 1 (1.9) | 0 | 5.895 | 3.811 | 9.118 | *<.0001* |
| ***General characteristics and treatment during the 1st 48h of ICU admission*** | | | | | | |
| Age, median [IQR] | 55.7 [46.9 ; 62.7] | 62.1 [53.9 ; 67.6] | **1.033** | 1.006 | 1.061 | *0.0172* |
| Female, n(%) | 11 (20.8) | 9 (21.4) | **1.184** | 0.566 | 2.477 | *0.6529* |
| Obesity, n(%) | 23 (43.4) | 14 (33.3) | **0.658** | 0.345 | 1.255 | *0.2040* |
| Diabetes, n(%) | 11 (20.8) | 14 (33.3) | **1.666** | 0.876 | 3.169 | *0.1199* |
| Chronic diseases, n(%) | 21 (39.6) | 25 (59.5) | **1.776** | 0.958 | 3.291 | *0.0681* |
| SAPS 2*, median [IQR] | 29 [22 ; 45] | 39.5 [29 ; 62] | **1.012** | 1.000 | 1.025 | *0.0476* |
| SOFA score*, median [IQR] | 5 [4 ; 7] | 7 [5 ; 10] | **1.192** | 1.087 | 1.306 | *0.0002* |
| C-reactive protein*, median [IQR] | 111 [64 ; 210] | 149 [86 ; 230] | **1.002** | 1.000 | 1.005 | *0.0884* |
| Steroids, n(%) | 31 (58.5) | 30 (71.4) | **1.387** | 0.709 | 2.712 | *0.3389* |
| Renal replacement therapy**, n(%) | 6 (11.3) | 9 (21.4) | **1.674** | 0.800 | 3.502 | *0.1710* |
| Vasopressors**, n(%) | 19 (35.8) | 22 (52.4) | **1.591** | 0.868 | 2.917 | *0.1328* |
| Ventilation status** |  |  |  |  |  |  |
| Mechanical ventilation with PEEP>10 cmH2O, n(%) | 10 (18.9) | 17 (40.5) | **1.992** | 1.035 | 3.834 | *0.0391* |
| ECMO, n(%) | 4 (7.5) | 6 (14.3) | **1.897** | 0.757 | 4.755 | *0.1719* |
| Antibiotics**, n(%) | 44 (83) | 39 (92.9) | **2.225** | 0.687 | 7.207 | *0.1821* |

*Legend:*

NB**: for time-dependent covariates, values of the covariates during the first 48 hours are given in column 2 and 3 for information.**

*NB2: only one patient was colonized with carbapenemase-producing Enterobacterales. The variable was not tested statistically..*

*SAPS 2: Simplified Acute Physiology Score ; SOFA score: Sequential Organ Failure Assessment score ; OTU: Operational Taxonomic Unit ; B/F ratio: Bacteroidetes / Firmicutes ratio ; ECMO: Extracorporeal Membrane Oxygenation; HR Hazard ratio on the final model ; 95% CI: 95% confidence interval of the adjusted HR; (*) at admission ; (**) during the first 48h in ICU ; (***) abundance
# Antifungal treatment active against Candida includes: IV polyenes, azoles and IV candins.*

*# Antibiotic treatment active against Enterococcus/MRSA includes: vancomycin, daptomycin and oxazolidinones.*

*# Antibiotic treatment active against anaerobic bacteria includes: nitroimidazoles, imipenem, meropenem, clindamycin, piperacillin/tazobactam and amoxicillin/clavulanate.*

**Additional file 1: Figure S1**. Quantitative culturing on agar plates. CFU: colony-forming unit.

**Additional file 1: Figure S2**. Pie-chart of the distribution of *Enterococcus* spp. and *Candida* spp. species obtained by culture of all the rectal swabs (A) and oropharyngeal (B) swabs.
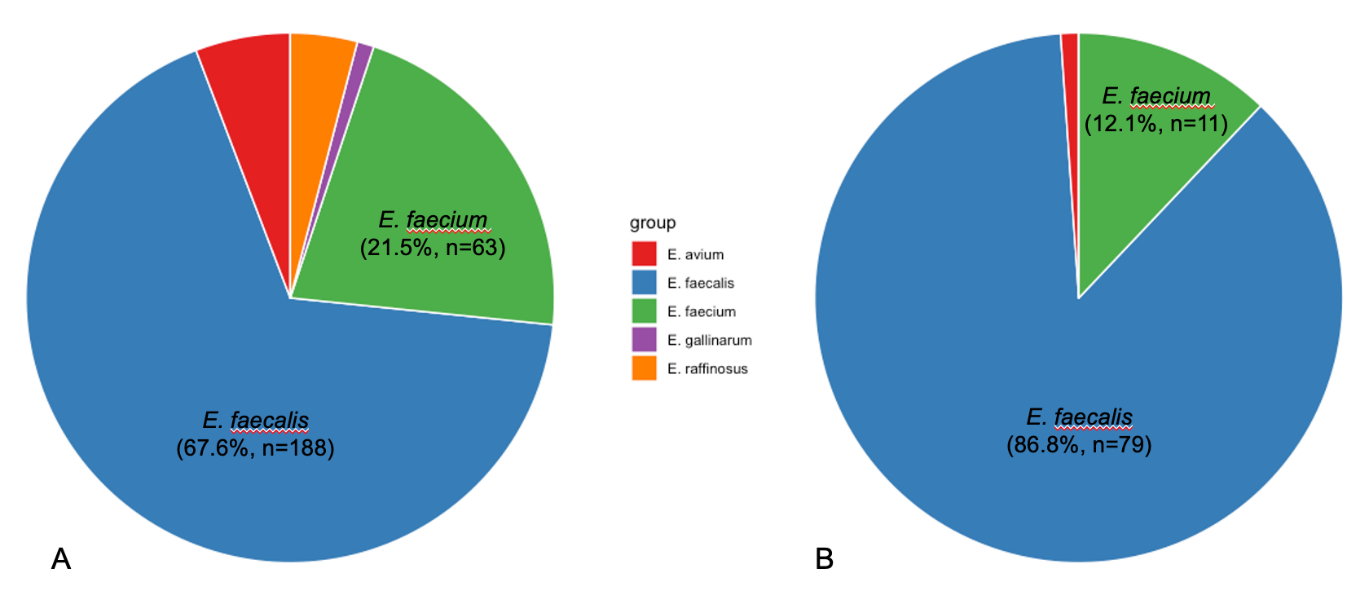


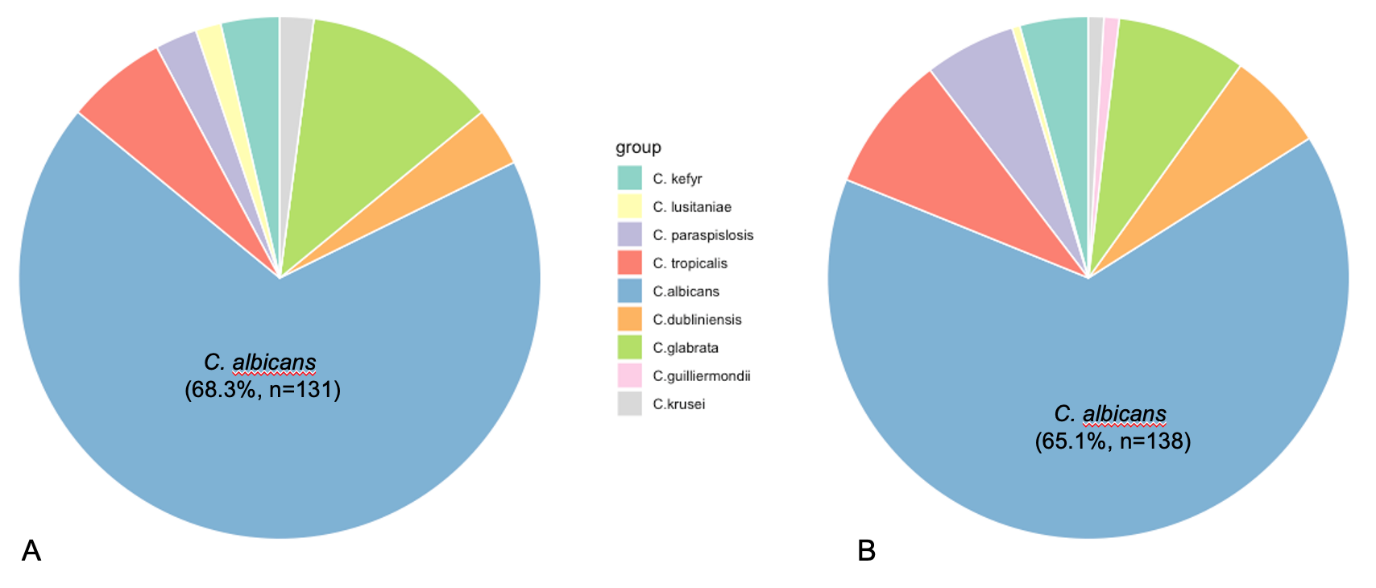


**Additional file 1: Figure S3**. Dot-plots of the intestinal *Enterococcus* spp. and *Candida* spp. with regards to intestinal richness (genus level), Shannon and inverse Simpson indices. The Pearson correlation test was used (with the intestinal concentrations being considered as continuous variables).
